# Partial Dislocations; Consecutive and Muscular Affections of the Shoulder Joint

**Published:** 1859-04

**Authors:** Alfred Mercer

**Affiliations:** Syracuse, N. Y.


### BUFFALO MEDICAL JOURNAL

AND

### MONTHLY REVIEW.

**VOL. 14.** APRIL, 1859. **NO. 11.**

### ORIGINAL COMMUNICATIONS.

ART. I.—*Partial Dislocations; Consecutive and Muscular Affections of the Shoulder Joint.* By Alfred Mercer, M. D., Syracuse, N. Y.

Case I. Oct. 12th, 1856, was called to attend Mrs. B. in her first labor; aged 24, nervous temperament, spare habit, lax fibre; had suffered from chorea in childhood, and recently, from what herself and friends termed rheumatism. In the last throes of labor, convulsions set in; after bleeding, spontaneous delivery soon took place, but convulsions followed in a few minutes, for which she was again bled, and a calomel purge administered. When consciousness was restored, in the night, she complained of pain and soreness about the right shoulder, and continued to do so on the following morning. While her linen was being changed, we handled the arm, and saw and examined the joint; there was no swelling or deformity to be observed, and the elbow was in a natural position, but any movement of the arm increased the pain in the shoulder.

The patient had a slow getting up. For two weeks she could not be raised in bed without a sensation of faintness. The pain continued in the shoulder, intermitting with pain in the head, about as often, and as severe in the head as in the shoulder, which was regarded as neuralgic or rheumatic. In the interval, I examined the shoulder two or three times, and found nothing wrong about it; used an anodyne liniment, and left Dover's powders for the pain, but the suffering was not thought sufficient for their admintration.

Nov. 5th, twenty-four days after labor. I had not seen the patient for several days. I was requested to call and examine the shoulder, for they had just discovered that it did not look like the other, and feared it was out of place.

By this time the patient had so far regained her strength as to be able to sit up a little in bed. On examining the joint, I found the head of the os humeri drawn forward, and, perhaps, a little downward, against the outside of the coracoid process. The head of the bone was prominent in front; the whole shoulder was thinner than the other, and flattened behind; the spine of the scapula, and the acromion, slightly projecting; the point of the shoulder tender to the touch; the arm in a natural position. The deltoid, the infra and supra spinatus muscles, appeared to be atrophied. The head of the humerus seemed to rest on the anterior edge of the glenoid cavity, and against the outer side of the coracoid process; and could it be carried backward one-fourth, or three-eighths of an inch, it would seem to be in place.

On the following day, the patient under chloroform, assisted by Dr. Pease, the head of the humerus was restored to its normal position without difficulty; a pad was placed in the axilla, and the arm brought well forward across the chest and secured by a roller, but still the shoulder had a flattened appearance behind, owing to the atrophied state of the muscles; and from their consequent feeble action, the head of the humerus did not remain well in place, which was again drawn forward, as I suppose, by the superior power of the pectoralis major; but I hoped time and use would restore the antagonizing power, and give perfection to the joint. In this, however, I was to a greater or less extent disappointed.

In February following, I was prosecuted in the case for mal-practice. I learned that Drs. Shipman, Hoyt, and others, had examined the shoulder. In conversation with these gentlemen, they both agreed with me in relation to the position of the head of the humerus, and that the synovial membrane and ligaments of the joint had not been ruptured. Dr. Shipman thought, from the lax state of the muscles, if the patient would let him, he could restore the bone to position, with the unassisted effort of his hands. The friends of the patient claimed that there was no difference in the appearance of the shoulder now and before it was reduced, and that it had never been reduced; they were correct as to its appearance, and I, no doubt, was in an error in attempting to reduce it. The patient could move the arm backward and forward, but could not carry the elbow far from the side, or the hand behind her or to the head, and the point of the shoulder was still tender, but there was less pain in the joint.

Fifteen months after her first labor she passed through a second without difficulty; the accoucheur arrived just in time to receive the child with his hands. Without any particular debilitating cause, the patient was very feeble; and a few days after confinement, was still further reduced by flooding. About this time she had retention of urine, calling for the use of the catheter, and also had what she and her friends called "faint spells," attended with muscular spasms, and sometimes, with frothing of the mouth. In one of these "spells," brought on by mental excitement and noise and confusion in the house, she unexpectedly died, and was buried without the body being examined.

Since the death of the patient, I have learned she had several similar attacks during the interval of the two labors, in one or more of which, the family were summoned to see her die; and that the arm was more useful than pretended.

We might inquire as to the cause and time of this displacement, and correctness of the diagnosis. The diagnosis is confirmed by Drs. Shipman, Hoyt, and Pease, whose opinions will hardly be questioned. If the displacement occurred at the time of the convulsions, it is strange the deformity, which was afterwards very plain to be seen by the attendants even, who first discovered it, should have been overlooked for twenty-four days by these self-same attendants; saying nothing about my own examinations of the case, and then the muscles were atrophied, which was not a momentary work from any cause, but a work of time. And we believe the displacement was a work of time, coming on gradually as the posterior muscles of the shoulder gradually wasted away and lost their antagonizing powers against the pectoralis major.

We have numerous examples of this loss of antagonizing power from paralysis, and atrophy, or from permanent spasms of one or more muscles or sets of muscles, as in paralysis of one side of the face, strabismus, club foot, wry-neck, lateral curvature of the spine, drooping of the wrist in lead poisoning, and in some forms of anchylosis of the joints.

We know, too, that convulsions themselves frequently produce luxations. Prof. Hamilton relates the case of an epileptic, (Trans. N. Y. S. M. S., 1855, p. 40,) in which the shoulder was frequently dislocated forward, and which he failed to reduce in a prolonged effort, but which the muscles themselves reduced the following night. I have seen an epileptic, who frequently had the humerus thrown into the axilla; and I am acquainted with a lady of lax fibre, who has many times been awakened from her sleep with the pain of a dislocated shoulder from morbid muscular action; and recently the newspapers have related of luxation of the shoulder from sneezing; and a case is on record, where the patient could luxate the shoulder at will, by muscular action. (Cooper's Dict., new edition.) And then, there are numerous cases of spontaneous luxation, both of the hip and shoulder-joint, on record, from relaxation of ligaments, spasms, and paralysis of muscles, and obscure rheumatic affections of the joints, and, no doubt, congenital luxations depend upon the same class of causes.

Mr. Stanley reported several of these cases in the London Lancet for 1840; and of these luxations, Mr. Erichsen, (Science and Art of Surgery, p. 249,) remarks: "The head of the bone slips out of the articulation without any well marked signs of disease about the joint, and certainly without any previous destruction of it." In these cases, there is either a paralytic condition of the capsular muscles, as has been observed several times in the shoulder, the deltoid having become paralyzed, and thus allowed the bone to slip out of place; or, as has been noticed in the hip, obscure rheumatic or neuralgic pains have been seated in the joint.

This dislocation may not be confined to one joint, but may affect several. Thus, some time ago, there was a case in University College Hospital, in which both shoulders and hips were dislocated spontaneously. In many cases it occurs suddenly, and often without pain to the patient, the deformity of the limb attracting attention to the accident, though in others it has been preceded by rheumatic affections of the joint. The treatment of these cases, he says, "is not very satisfactory." Reduction in many, cannot be accomplished, but in others, it may be effected readily enough, though the bone cannot be fixed in the joint, out of which it slips again.

Case II. Feb. 2d, 1857. P. L., aged 24, a strong, muscular, laboring man, in a drunken scuffle, dislocated his left shoulder into the axilla, which was successfully reduced the same evening by Dr. Pease, through whose politeness I saw the case, three weeks after the accident. On examining the shoulder, it was seen to be thinner than the other, flattened behind, and fuller in front, the spine of the scapula and acromion prominent, the deltoid, and the infra and supra-spinatus muscles atrophied, and the head of the os humeri drawn forward against the outside of the coracoid process. The patient could move the arm forward and backward, but could not raise it from the side. Under the influence of chloroform, the bone was readily reduced, but directly returned to its former position, and all efforts to retain it in place proved abortive. Seven months after, since which time I have lost sight of the patient, the movements of the arm had improved, but the shoulder presented the same general appearance.

Case III. Mrs. B., a well developed woman, of full habit, aged 56, seven years since, was thrown from a carriage, dislocating her right shoulder, which was reduced a short time after the accident, but the shoulder was painful, and tender to the touch, and almost useless for months after. She could carry the arm forward and backward, but could not raise it from the side, or carry the hand behind her, or raise it to her head, for fourteen months. She has gradually gained better use of her arm, bnt now, July, 1858, she cannot raise the elbow from the side more than half-way to a horizontal position without assistance, but with assistance, the arm may be carried into any position, without pain or resistance. Measurement shows no appreciable difference in the size or length of the arm, or size of the shoulder; but the point of the shoulder is still tender to the touch, is prominent in front, and correspondingly flattened behind. The head of the humerus appears to rest against the outside of the coracoid process, but the fullness of habit obscures the diagnosis, compared with the other cases. Several doctors, at different times, have examined the shoulder; some have said it was not properly reduced, and advised a suit for mal-practice.

I examined the shoulder again in November last; it presented the same general appearance, although the patient was much thinner in flesh from recent sickness. Some six weeks previous to this examination, in a sudden and thoughtless effort to raise the arm above the head, the muscles unexpectedly obeyed the will; since which time she has had perfect use of it, though the deformity still remains. She thinks she felt or heard a snap when the arm went up, but it was followed by no pain, soreness, or swelling.

In all other cases, the head of the humerus seemed to settle well into the glenoid cavity, when the elbow was carried across the chest.

In view of surgical literature, it might be questioned whether these are really partial dislocations of the shoulder-joint; but what are they if they are not? The head of the humerus is not in its natural position in the glenoid cavity, neither is it thrown out of it, which would seem to answer to a common-sense idea of a partial, in distinction from a complete luxation, where the articulating surfaces are wholly removed from each other.

Surgical writers would seem to have paid but little attention to this class of affections. Gibson, McClellan, S. Cooper, Liston, and others, as far as I am acquainted with their writings, have passed the subject over in silence.

Mr. Druitt has a short article on Partial Dislocations of the Shoulder-joint, but what he says on the subject is evidently compiled from Sir Astley Cooper, who reports four cases under the head of "Partial Dislocation of the os Humeri," in his work on fractures and dislocations, p. 358, N. E. The head of the humerus would appear not to have been in the same position in all of Sir Astley's cases; and he speaks of them, too, as depending altogether on a traumatic cause; yet a moment's reflection on his account of the first case will show it to have been muscular in its pathology. He says: "By extension of the shoulders backward, I at last brought the head of the bone to the glenoid cavity, but it directly again slipped forward as the extension ceased." The slipping forward, as the extension ceased, can be attributed to nothing but morbid muscular action.

Of his second case, he says: "The os humeri rests against and under the coracoid process," and of his third and dissected case, he says: "The os humeri was situated under the coracoid process, which formed the upper part of the new glenoid cavity."

Mr. Erichsen, (Science and Art of Surgery, p. 245,) expresses some surprise, that Sir Astley Cooper should have described this as a partial dislocation, when the head of the humerus was apparently completely thrown from the glenoid cavity; and remarks, that these "dislocations appear to point to a form of injury of the shoulder-joint, which of late years has been specially described by the French surgeons, as a variety of dislocation downward; that form of displacement, indeed, which by Boyer, has been described as the *dislocation inward;* by Malgaigne, the *subcoracoid luxation*, and by Velpeau, as the *subscapular dislocation*, in which the head of the humerus is placed in front of the neck of the scapula, and under the subscapular muscle."

Mr. Erichsen would confine the term partial dislocation to that form of displacement described by Mr. Soden. (See Sir A. Cooper's Fractures and Dislocations note to N. E., p. 361, and Mr. Smee, Ranking's Abstract, 1847, p. 102,) cases which these gentlemen have dissected, and found the long head of the biceps either ruptured or thrown from its groove, and the head of the humerus drawn forward and upward against the acromion.

Mr. Miller describes two forms of partial dislocation of the shoulder-joint. One in which the long head of the biceps is ruptured or thrown from its groove; the other, he calls a "subluxation on the coracoid process," and says a partial displacement may take place in this direction. There is slight flattening of the shoulder, with a corresponding degree of displacement beneath the acromion, and the head of the bone is felt, and seen projecting on the coracoid process. Reduction is beset with no difficulty; in fact, the manipulations required for a diagnosis generally succeed in effecting replacement. The accident is rare."

In this, Mr. Miller evidently refers to a partial luxation from an external injury. He says the accident is rare, and no doubt it is, if it occurs at all from such a cause, and its reduction is beset with no difficulty. He would seem to have no idea of a partial luxation from a muscular cause.

On this subject, Prof. Hamilton writes me, that "the subluxation" of the shoulder-joint, "as an immediate result of an accident, and continuing, (as described by Sir Astley Cooper,) has been denied by almost every surgical writer since his day; Malgaigne says it is impossible, and so I think, also." Or, to quote Malgaigne: "As for myself, the question is definitely settled, that there cannot be a traumatic luxation which retains the head of the humerus at the external side of the coracoid process, but there is a subluxation of the humerus, owing to inflammation of the joint, and retraction of the ligaments. (Malgaigne's Dislocations, p. 567.)

Bransby Cooper, better perhaps than any other English writer, would seem to comprehend the nature of these affections. Speaking of partial dislocations forward, he says, (p. 288, Lectures on Surgery): "I have no experience of this accident, and believe it more frequently the effects of disease in the joint, or constitutional derangement, than of external injury."

Of the different luxations to which the shoulder-joint is liable, he says: "There is a fourth luxation spoken of by Sir Astley Cooper, termed a partial dislocation, in which the articular surface of the humerus glides upon the inner edge of the glenoid cavity, and rests upon the base of the coracoid process of the scapula. In such cases, I believe the capsular ligament is not torn through, and that the partial displacement depends upon the relaxation of the muscles from some constitutional cause, and the difficulty which occurs is less in reducing the dislocation than in maintaining coäptation."

Mr. Liston says, (article Anchylosis): "Often, too, a loss of muscular power, either by sloughing, or wound, or paralysis, on one side of the joint, by destroying, as it does, the balance of power which naturally exists, will bring on this 'muscular' form of anchylosis. The sound muscles are no longer opposed by their antagonists, pull the limb into an unnatural position, and, as there is no power to overcome this influence, they keep it contracted."

And speaking of muscular atrophy, Mr. Paget says, (Surgical Pathology, p. 90): "Besides the general atrophy of muscles, a similar affection occurs sometimes as a primary or spontaneous affection of one or more muscles.

We find, sometimes, one of the muscles of an extremity, or of the back, thoroughly atrophied, while the others are healthy, and no account can be given of its failure." * * *

"Mr. Mayo has recorded two cases of apparent spontaneous atrophy of the muscles of the shoulder, in which, a few weeks after severe pain, but no other sign of acute inflammation, all the muscles about the shoulder became simply, but exceedingly, atrophied."

Rokitansky, (Ranking's Abstract, 1857, pp. 1, 41,) relates the case of a laborer, who, after allowing his wet clothes to dry on him, had severe pain in his left shoulder, but no swelling. He could not raise his arm, and after a week, the shoulder was found wasted, and as the pain subsided, the wasting advanced, till the deltoid, supra-spinatus, infra-spinatus, and teres muscles seemed completely absorbed.*

These affections of the shoulder-joint are of interest to the surgeon, both in a pathological, and in a legal point of view—for a prosecution was commenced in one case,† and it was a subject of earnest conversation in the other two—that their nature should be understood.

I have presented what was at hand to illustrate their pathology; and at the risk of being tedious, I wish still further to continue my observations, in somewhat of a general way, on the obstacles to reduction, and the condition and means by which reduction is effected and maintained.

But first a word or two on the anatomy of the shoulder-joint; and we find what is quite to our purpose in the Dublin Dissector:

"The deltoid, and the four capsular muscles, supra, and infra-spinatus, teres minor, and subscapularis, which have been just described, are of great use to the shoulder articulation; the head of the humerus is so large, the glenoid cavity so superficial, and the capsular ligaments so loose and long, that but for these muscles, the bones could not remain in opposition; hence, in case of paralysis of this region, the joint becomes elongated and flattened, and a *partial dislocation exists.*"

Tonic muscular contraction, the projecting lip of bone surrounding the articular cavity, and the difficulty of returning the head of the dislocated bone through the ruptured capsule of the joint, are regarded as the great

---

*On this and kindred subjects see New York Journal of Medicine, vol. 11th, new series, p. 427, and William Roberts on Wasting Palsy.

† The suit has never been tried. I sued the party for my fees in justice court, and when we were ready for trial, the whole matter was agreed to be referred, which has not yet been done, and the patient is dead.

obstacles to reduction. But muscular contraction, no doubt, is the great obstacle, and it is also the equally great reducer of dislocations, the surgeon only acting to bring the axis of the dislocated bone in the line of direction of the articular cavity. In a downward dislocation of the shoulder, the muscles are acting to draw the head of the humerus upward and inward. The surgeon counteracts these forces by extension downward, and with the heel in the axilla, as a wedge, or fulcrum, he carries the head of the bone outward, and when these counter forces have brought the head of the humerus below and to the outer side of the glenoid cavity—the muscles still acting in the same direction—and as the head of the bo e glides over the under rim of the glenoid cavity, the muscles, with a unanimity of action, and with a greater power than the operator can possibly exert, or, perhaps, greater than they have exerted at any time, for any other purpose, the bone is restored in an instant to its normal position, and frequently with such force, in spite of the extension, as to produce an audible concussion.

Mr. Skey remarks on this subject:

"I consider that the muscles are the immediate agents of reduction, and not the surgeon, whose entire duty consists in placing the bone in a position to give them the opportunity of displaying this harmony of action, and of exerting a power even beyond that of the mechanical agent of extension. I believe if we bring the bone sufficiently down, and place it in the neighborhood of the articulation, the muscles will replace it with as much ease as that which originally dislolocated it."

Now if these views are correct, and I believe every surgeon will admit they are, in relation to the part played by the muscles in the reduction of dislocations, such a thing as a partial dislocation of a ball and socket-joint, the muscles retaining their normal action, is impossible, that could not be readily and permanently reduced.

If from accident, the head of the os humeri should be thrown on the rim of the glenoid cavity, it would rest there as on a pivot; the muscles, healthy, would still be acting to draw the bone upward and inward, toward the body, from both before and behind, and any considerable movement of the joint, would so disturb the balance of the rounded head of the humerus on the articular rim, that it would be either drawn completely from the glenoid cavity, or back into it.

Where the socket is as shallow as in the shoulder-joint, it can have but little influence in retaining the head of the humerus in position; and where the movements are so free and varied, the capsular ligaments must be lax when compared with other joints, to admit of such freedom of motion; and where the strength of the joint depends so much on its muscles, when, as part, or all of them, are disordered in action by paralysis, atrophy, or morbid contractions, we can readily understand how they can produce distortion of the joint.

A partial dislocation of the hip-joint is a thing almost wholly unknown; although it is subject to spontaneous luxation from muscular spasms, rheumatic, and other diseases of the joint, and from relaxation of its muscles and ligaments.

All the partial luxations of the shoulder occur in the direction of its overhanging and protecting processes—the acromion and coracoid—and the question arises, whether these luxations would not be complete, as in the hip-joint, whatever might be the cause, were it not for these obstacles. We have no partial dislocations of the shoulder-joint downward or backward; they are either complete, or not at all, in these directions.

The most powerful muscles of the shoulder-joint, no doubt, are the pectoralis major and the deltoid; the one to draw the humerus forward, the other upward; and when the posterior muscles lose their antagonizing power, there is nothing to resist the pectoralis drawing the humerus forward against the coracoid process.

All muscles are opposed by some force, in their contractions. When they have not, their extremities, within certain limits, continue to approximate each other—shortening of the thigh, in fracture of the femur, is a familiar example—and when the posterior muscles of the shoulder lose their antagonizing power, the ligaments of the joint, with the glenoid cavity, tipped as it is with its articular cartilage, would offer but feeble resistance to the continued strain of the pectoralis major; and the head of the humerus would be drawn forward against the coracoid process, almost as readily as the eye would be turned inward or outward, by the paralysis or spasms of the recti muscles of the eye in strabismus. And if this morbid action should still go on, (as it did in a case occurring in the practice of Dr. Campbell, of Livingston county, N. Y., and reported by Prof. Hamilton in the Trans. N. Y. S. M. S., 1855, page 42,) the head of the bone would most likely, as in this case, descend on the outside of the coracoid process, then glide under it, and again mount upward on the sternal side of the coracoid process, under the clavicle, rather than ride directly over it.

The deltoid is powerful in its upward action, and is chiefly antagonized by the long head of the biceps; and when this tendon is ruptured, or thrown from its groove, we have the only true partial dislocation of the shoulder from a traumatic cause, as admitted and described by Soden, Smee,

Malgaigne, and Erichsen; and a force capable of rupturing the long head of the biceps, would, no doubt, render the luxation complete, were it not prevented by the acromion process, which could not take place in this direction, without a fracture of that process, and extensive laceration of muscles.

With these facts and considerations before us, the immediate cause of the affection of the shoulder-joint, to which I have directed your attention—call it what we may—would seem to depend on morbid muscular action, the anterior overpowering the posterior. But the true pathology lies farther back than this, in the constitution, or in the nervous system, or local inflammation, which may have degenerated the muscular fibre, or interrupted its nutrition.

In case No. 1, the shoulder, no doubt, received some injury during the convulsions; most likely a strain, for I remember to have seen one of the arms, which, I cannot say, in an awkward position, and cautioned the attendants not to make too great an effort to restrain the movements of the patient; but the nerve fibres or nervous centres may have been at fault, or a simple overstrain of the muscles from the convulsions alone may have been the cause of the paralysis and atrophy, for a single overstrain of a muscle is sometimes followed by loss of power. (Barclay's Medical Diagnosis, p. 179.) Paralysis of the bladder from distension of urine is a familiar example. And then the previous and subsequent history of the patient, the chorea of childhood, the rheumatism, the shifting pain from shoulder to head, the retention of urine after her second confinement, and the convulsions, with frothing at the mouth, in the one of which she died, all point to deep-seated nervous and constitutional disease.

From what has been said, we should hope something like a rational pathology might be made out for the other two cases of partial displacement following the successful reduction of a complete luxation.

Mr. Druitt, no doubt, had something like these affections in view, in saying: "Injuries of the shoulder-joint are liable to be followed by various obstinate and intractable affections. Sometimes the deltoid wastes away, owing probably to injury of the circumflex nerve."

If such a thing is possible, from a traumatic cause, as a partial luxation of the head of the humerus, where the bone is retained external to the coracoid process—save the upward luxation from rupture of the long head of the biceps, or its being thrown from its groove—the muscles uninjured, its reduction, in the language of Mr. Miller, can be beset with no difficulty; but when the displacement depends on morbid muscular action, as we think we have shown it always must, retentive reduction is beset with almost unsurmountable difficulties. In fact, the conditions of successful reduction, as laid down by Mr. Skey, are more than complied with in these partial luxations, for the bone is not only in the neighborhood of the articulation, but is in partial contact with it.

In case second, all contrivances to retain the head of the bone in position, utterly failed, and I believe all attempts at forced reduction are entirely useless. Simple reduction, and retention of the head of the humerus, in its normal position, could, of itself, be of no service, unless power could be given to the posterior muscles and ligaments of the joint, to retain it there. We should hardly expect a successful, retentive reduction in a case of lateral curvature of the spine, yet the two affections would seem to arise from the same cause, loss of tone in muscular fibre, and we believe the same principles of treatment are applicable to both affections. Constitutional treatment, to give tone and vigor to the system, with frictions, shampooing, electricity, and exercise, or any other means that would be likely to give tone to muscular fibre and increase its nutrition, would seem to be the most rational treatment, and offer the greatest hope of success; but this treatment would be of little service, should the atrophy be dependent on lesions of the nervous trunk or centres.

